# Knowledge Domain and Emerging Trends on Echinococcosis Research: A Scientometric Analysis

**DOI:** 10.3390/ijerph16050842

**Published:** 2019-03-08

**Authors:** Xingming Ma, Lifeng Zhang, Jingqiu Wang, Yanping Luo

**Affiliations:** 1 Department of Immunology, School of Basic Medical Sciences, Lanzhou University, Lanzhou 730000, China; maxm@lzu.edu.cn (X.M.); zhanglf@lzu.edu.cn (L.Z.); wangjq@lzu.edu.cn (J.W.); 2 Key Lab of Preclinical Study for New Drugs of Gansu Province, Lanzhou 730000, China

**Keywords:** echinococcosis, scientometrics, CiteSpace, visualization analysis, emerging trends

## Abstract

The echinococcosis of humans and animals is a chronic helminthic disease caused by the larva of genus *Echinococcus* tapeworms. It is a globally distributed disease which is an important socioeconomic and public health problem in many low and middle-income countries. This research aimed to firstly quantitatively analyze the publications with bibliometrics software and evaluated the hot topics and emerging trends of echinococcosis research from 1980 to 2017. A total of 7688 references on echinococcosis research were retrieved from the Web of Science Core Collection database. Then the reference was analyzed with CiteSpace software to make the knowledge network maps. The largest cluster (#0) with 83 members was cystic echinococcosis, and cystic echinococcosis, mebendazole, antibody and transmission were the four keywords with the strongest citation bursts in the echinococcosis research field. Furthermore, cystic echinococcosis, chemotherapy and immunodiagnosis, management of definitive and intermediate host are the top four research hot topics and emerging trends in the echinococcosis field. This research presents an insight into the echinococcosis field and valuable visualizing information for echinococcosis researchers to detect new viewpoints on cooperative countries/institutions, potential co-workers and research frontiers.

## 1. Introduction

Human and animal echinococcosis (or hydatid disease) is a chronic cyst-forming and neglected zoonosis caused by infection with the larval stage of the genus *Echinococcus* (*E.*) [1,2]. Despite global scientists making maximizing efforts to minimize helminthic infections of *E.* in the past twenty years, numerous human cases of such diseases are still reported worldwide [3,4,5]. Echinococcosis is the main endemic in sheep-raising and/or cattle farming areas of South America, Australia, in the Baltic region, the Middle East, the Mediterranean region, Africa, and Central Asia including China. However, along with increased tourism and travel over the whole world, it can be found anywhere, even in developed countries [6,7,8]. The most frequent clinical forms of echinococcosis are alveolar echinococcosis (AE) caused by *E. multilocularis* and cystic echinococcosis (CE) caused by *E. granulosus*. In particular, CE probably accounts for more than 95% of all echinococcosis and the hydatid cyst is the most common performance [1,2,9,10].

During its life cycle, the adult of genus *E.* resides in the small bowel of definitive hosts such as dogs or other wild carnivorous mammals. Then, the eggs released by gravid proglottids are passed in the feces. The common intermediate hosts are sheep, cattle or other ruminants [2,10,11]. These eggs are ingested by intermediate hosts and oncospheres are released in the small bowel. The oncospheres migrate to the liver, or, less commonly, lodged in other organs. At the organ site, the embryos either develop into hydatid cysts or die [2,10,11]. 

Humans become accidentally infected by eating foodstuffs or drinking water contaminated with *E.* eggs or by indirect or direct contact with infected definitive hosts [2,10,11]. The disease in humans involves the development of a fluid-filled hydatid cyst, which generally localizes in the lungs and/or liver [1]. It continues to be an important cause of mortality and disability in many pastoral areas of the world [1,6,7,8]. It is increasingly recognized as a major public health problem and economic burden concern in many regions, particularly in low-income countries [10]. The treatment means of echinococcosis are still difficult since surgical operation cannot fit the needs of all patients, and drugs can lead to serious adverse events as well as resistance [12,13]. It is serious in nature, the difficulty of vaccination and treatment make it an important topic of investigation, including China. 

Bibliometrics, a quantitative statistical analysis tool, are frequently used in the fields of publications and information science to provide quantitative analyses of academic literature [14]. The method of bibliometrics has been used to assess patterns in co-citation, authors, journals, institutions, countries, and keywords associated with specific document types in many research fields [15,16]. Nevertheless, a particular bibliometric analysis of global echinococcosis research from 1980 to 2017 has not yet been carried out. 

CiteSpace software, a visualization analyzing tool, was created by Professor Chaomei Chen in early 2004 [17]. CiteSpace software is characterized by co-occurrence network maps of countries, institutions, authors, keywords, and subject categories and co-citation networks of cited authors, cited references, and cited journals to analyze the literature gained from the databases of Web of Science [18,19,20,21,22,23].

The Web of Science Core Collection database is the most frequently used source of scientific information [18,19,20,21,22,23]. In the current study, in order to investigate the hot topics, trends and situation in the global echinococcosis research field, a total of 7688 publications from the establishing database time of 1980 to 2017 was obtained from the Web of Science Core Collection databases. Then, the centrality and network of cited journals, cited references, cited authors, keywords and citation bursts of reference were firstly analyzed in the visualization pattern by CiteSpace, which is helpful to gain more complete and accurate information about echinococcosis research domains.

## 2. Materials and Methods 

### 2.1. Data Retrieval Strategy

We carried out a comprehensive literature retrieval using string index words about echinococcosis as follows: “echinococcosis or hydatidosis or hydatid disease or *Echinococcus granulosus* or *Echinococcus multilocularis* or protoscolex or protoscoleces or protoscolices.” The timespan for the search was from 1980 to 2018 (38 years, retrieved date 1 May 2018). All electronic searches were performed on the same day, 1 May 2018.

### 2.2. Data Collection

We collected the data for bibliometric analysis from the Web of Science Core Collection databases (WoSCCd) including SSCI, SCI-Expanded, CPCI-S, A&HCl, ESCI, CPCI-SSH, CCR-Expanded and IC. The WoSCCd is the most frequently used source of scientific information [18,19,20,21,22,23]. A document type was only article and web of science categories, or the language of article was not restricted. A total of 7775 bibliographic records (articles) was gained from the WoSCCd and then the data were analyzed with CiteSpace. The 7688 documents (articles) from 1980 to 2017 should be employed to investigate the knowledge domain and development trends of the echinococcosis research field. 

### 2.3. Documents of Analysis Tool

CiteSpace software is very useful in generating knowledge maps and conveniently used to perform a bibliometric analysis of countries, institutions, authors/co-cited authors, journal/co-cited journal, keywords and co-cited references [17,24]. Three central results of citation bursts and centrality scores, heterogeneous networks are detected by CiteSpace software. Three practical parameters are used to identify the nature of a research front, detect the emerging trends and abrupt changes in a timely manner [17]. In brief, the burst detection algorithm can be suitable to measure the sharp increasing of interest within a short time span [17,25]. According to citation burst terms extracted from bibliographic records, a present research front is distinguished in CiteSpace software. In CiteSpace, the normalized value of betweenness centrality is between 0 and 1 in the unit interval, and the nodes with high centrality scores are denoted by a purple ring in a visualized network [17,24,25]. The indicator of centrality is used to assess the literature importance and the level of each literature within a co-citation network partially [26]. The values of mean silhouette (S) and the modularity (Q) are two indicators used to assess the “overall structural properties” of the network. The value of modularity is more than 0.3 (Q > 0.3), which means that the network is significant. The value of silhouette ranges from −1 to 1. While the silhouette is more than 0.5 (S > 0.5), the clustering of the network map is rational and acceptable [17,24,25].

### 2.4. Parameter Setting of CiteSpace

According to the literature recommendation [17,24,25], the better parameter choices in CiteSpace with version 5.1.R8 were set as follows: time span (from 1980 to 2017), time slicing (1), term source (all selections: including title, abstract, author, keyword and author plus), node type (choose one at a time: countries, institutions, authors, cited-author, cited-reference, cited-journal, keywords, respectively), selection criteria (top 30% per-slice), pruning (pathfinder and pruning the merged network) and visualization (cluster view-static and show merged network). Meanwhile, a time span in a one-year slice was indicated with a different color in the network links and nodes (such as red, yellow, green and blue).

## 3. Results and Discussion

### 3.1. Analysis of Publication Outputs

**Figure 1:**
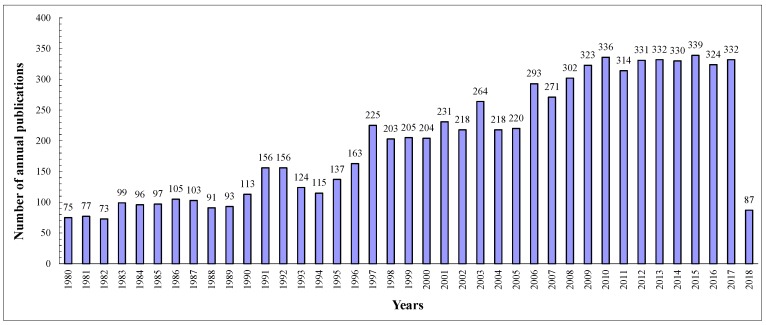
Number of annual publications on echinococcosis research from 1980 to 2018 in Web of Science Core Collection databases (WoSCCd). The period from 1980 to 1996 was the first stage with average annual outputs of 110 articles, while the period from 1997 to 2007 was the second stage with 232 articles and the third stage from 2008 to 2017 had 326 articles.

From 1980 to 2018 (retrieved date 16 May 2018), a total of 7775 publications of article type was obtained from the WoSCCd. The published article numbers on the echinococcosis research field increased from 75 in 1984 to 332 in 2017 (Figure 1). The number of published articles with a non-linear correlation increased over the studied period but with some exponential functions. Figure 1 showed that the studied period on echinococcosis could be divided into three stages. The period from 1980 to 1996 was the first stage, while the period from 1997 to 2007 was the second stage. The third stage from 2008 to 2017 was a speedy development period, and the average annual outputs of published articles were 326. Furthermore, the average number of published articles in the third stage was the highest among all the stages (e.g., 110 articles in the first stage and 232 articles in the second stage, respectively). 

The data showed that the science of echinococcosis, as an important and independent branch of medical discipline, was obtaining a great deal of attention and more echinococcosis research was being emphasized and performed. After 2008, the study on echinococcosis is booming. In 2008, several significant documents with higher centrality which accelerated the research of echinococcosis were issued. An example, “Multidisciplinary studies, systems approaches and parasite eco-epidemiology: something old, something new” by Giraudoux et al. [27], which initiated the multidisciplinary studying, proposed using eco-epidemiological approaches to confirm parasite transmission systems. Another example is “*Echinococcus ortleppi* and *E. granulosus* G1, G2 and G3 genotypes in Italian bovines” by Casulli et al. [28], and “Species identification of human echinococcosis using histopathology and genotyping in northwestern China” by Tiaoying et al. [29], which focused on the molecular genetic characterization of different strains of *E. granulosus*. Furthermore, the diagnosis and treatment measures of echinococcosis have been continuously improved through the efforts of global researchers.

### 3.2. Analysis of Country and Institution

**Figure 2:**
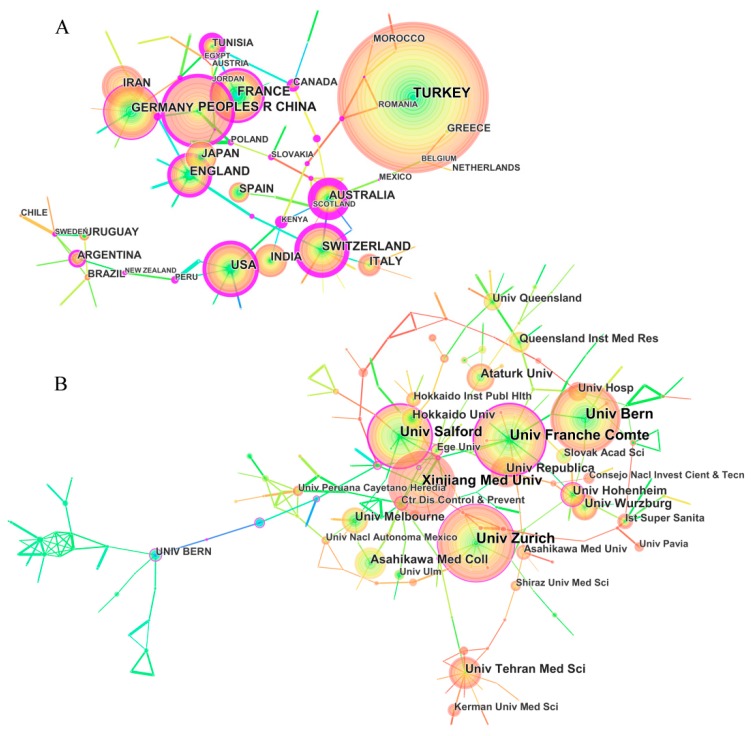
Network map of countries/regions (**A**) and institutions (**B**) that contributed to publications on echinococcosis research from 1980 to 2017. (A) The top ten countries/regions that contributed to publications on echinococcosis research were Turkey (1133), France (582), China (574), USA (521), England (485), Germany (482), Switzerland (480), India (376), Australia (356), and Spain (355). The top ten countries/regions in terms of centrality were Australia, Kenya, Tunisia, Switzerland, Canada, China, USA, Thailand, England, and France. (B) The top five institutions were Univ Zurich (German), Univ Bern (Switzerland), Univ Salford (England), Univ Franche Comte (France), and Xinjiang Med Univ (China). The top five institutions in terms of centrality were Univ Melbourne, Univ Salford, Univ Franche Comte, Ctr Dis Control & Prevent, and Murdoch Univ.

The 7688 article publications on echinococcosis research were contributed by 150 countries/ regions. A country/regions network map generated with the software of CiteSpace resulted in 84 nodes and 96 links with a mean Silhouette, S = 0.69 and modularity, Q = 0.79. The Q-value of countries network map was more than 0.70 (Q > 0.70), which denoted the nodes within the network are loosely assembled. This data showed that there were restricted collaborations between countries/regions (Figure 2A). The top 10 countries/regions contributing to publications on echinococcosis research from 1980 to 2017 are showed in a supplemental file. Turkey had the largest number of published articles (1133), followed by France (582), China (574), USA (521) and England (485). The top 10 countries/regions in terms of centrality (i.e. purple round in Figure 2A) were Australia (1.06), Kenya (0.87), Tunisia (0.66), Switzerland (0.61), Canada (0.58), China (0.56), USA (0.56), Thailand (0.55), England (0.44), and France (0.30). The analytical results in terms of publication and centrality showed that France, China, USA, England, Australia, and Switzerland were the major research powers in the echinococcosis field. 

The 7688 articles were distributed among 5402 research institutions. An institution network map generated with the software of CiteSpace resulted in 409 nodes and 340 links with a mean Silhouette, S = 0.24 and modularity, Q = 0.87. Since the Q-value of the institution network was more than 0.7, the nodes within the institution network are loosely assembled. Compared with countries, there is very little cooperation between the institutions (Figure 2B). The top five institutions were Univ Zurich (German), Univ Bern (Switzerland), Univ Salford (England), Univ Franche Comte (France), and Xinjiang Med Univ (China). The top eight institutions in terms of centrality were Univ Melbourne (0.24), Univ Salford (0.23), Univ Franche Comte (0.22), Ctr Dis Control & Prevent (0.19), Murdoch Univ (0.15), Yarmouk Univ (0.15), Univ Bern (0.14), and Univ Zurich (0.14). The analytical results in terms of publication and centrality presented that the major four research institutions were Univ Zurich, Univ Bern, Univ Salford, and Univ Franche Comte. Those data indicated that the advanced levels and techniques of echinococcosis research were in developed countries and institutions.

### 3.3. Analysis of Author/Co-Cited Author and Co-Cited Journals

**Figure 3:**
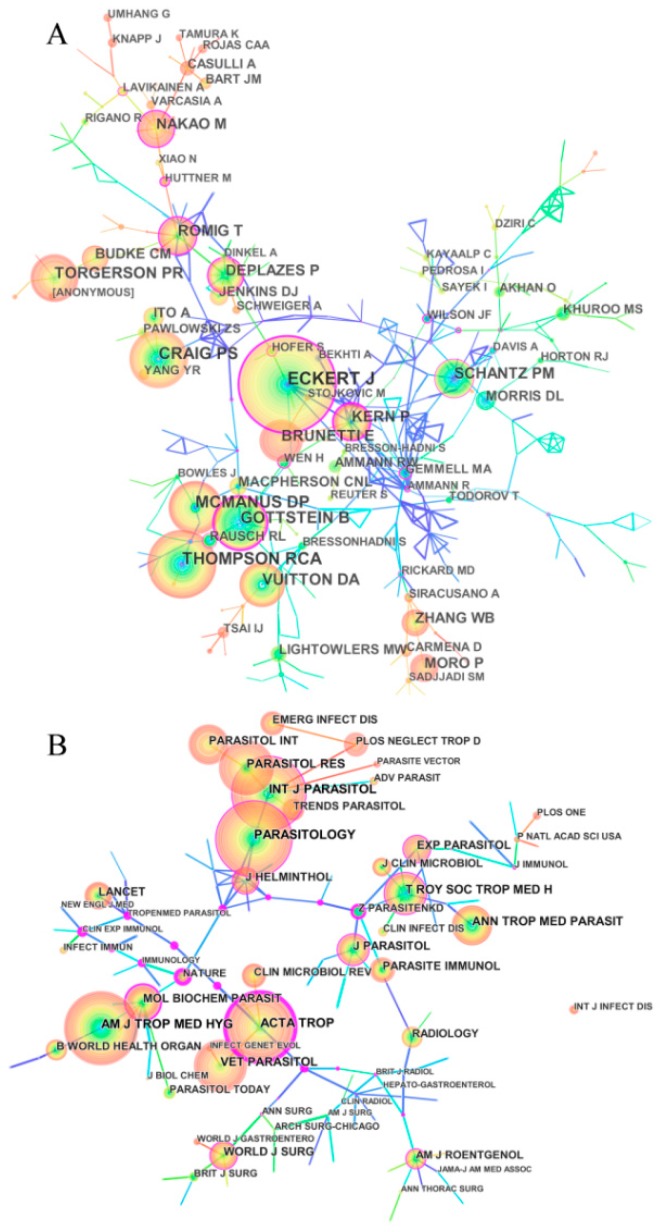
Co-cited authors (**A**) and cited journals (**B**) network map of publications on echinococcosis research from 1980 to 2017. (A) The top five co-cited authors were Eckert J (1508 citations), Thompson RCA (907 citations), Craig PS (787 citations), McManus DP (783 citations), and Gottstein B (751 citations) and the top five co-cited authors in terms of centrality were Beard TC, Nelson GS, Eckert J, Schwabe CW and Kern P. (B) The top five journals of publications were Vet Parasitol, Parasitol Res, Parasitology, Acta Trop and Am J Trop Med Hyg. The top five co-cited journals were Parasitology (2245 citations), Int J Parasitol (2202 citations), Am J Trop Med Hyg (2201 citations), Acta Trop (1846 citations) and Parasitol Res (1593 citations).

More than 22,445 authors contributed to the total number of publications. The top five productive authors were Craig PS (171 publications), Gottstein B (167 publications), Wen H (136 publications), Vuitton DA (123 publications), and Ito A (120 publications). Generating an author co-citation network map using CiteSpace resulted in 441 nodes and 550 links with a mean Silhouette, S = 0.45 and modularity, Q = 0.87 (Figure 3A). The size of the circles represents the amount of author co-citation and the line number between two circles suggests more collaboration between individual authors. The data indicated that many authors on echinococcosis research tended to cooperate with two or more core authors. 

CiteSpace detected the information on author citations and presented it through a network map. The network map of co-citation can provide information on influential research groups and potential collaborators and can help researchers to establish collaborations [17,24,25]. From Figure 3A, the top five co-cited authors were Eckert J (1508 citations), Thompson RCA (907 citations), Craig PS (787 citations), Mcmanus DP (783 citations), and Gottstein B (751 citations) and the top five co-cited authors in terms of centrality were Beard TC, Nelson GS, Eckert J, Schwabe CW and Kern P. An analysis in terms of centrality and co-citation counts revealed that Eckert J, Mcmanus DP and Schantz PM were “core strength” researchers on echinococcosis and their exploration had a substantial impact on the echinococcosis field. 

Eckert J worked at the University of Zurich (Switzerland) and studies the geographic distribution and epidemiology, treatment in animals (with a focus on chemotherapy), control and basic field of echinococcosis [30]. Thompson RCA worked at the Murdoch University (Australia) and studies the molecular epidemiology of parasites, the impacts of parasites on wildlife, and control measure of transmitting parasites [31]. Mcmanus DP worked at the Queensland Institute of Medical Research (Australia) and studies intermediary carbohydrate metabolism in protoscoleces, the epidemiology, treatments and control strategies [32]. 

In total, 1305 academic journals have published articles on echinococcosis research. The top 10 scholarly journals and cited journals in terms of co-citation counts and centrality on the echinococcosis research field are shown respectively in a supplemental file. Generating a co-citation journal map using CiteSpace resulted in 320 nodes and 970 links to detect the most substantial co-cited journal in Figure 3B. The network of cited journals had a mean silhouette, S = 0.31 and modularity, Q = 0.83. The size of the circles represents the co-citation frequency of each journal within a co-citation network. The analytical results in terms of citation frequency and centrality showed that the journal of Int J Parasitol, Parasitology and Acta Trop were the “core journals” on the echinococcosis research field.

**Figure 4:**
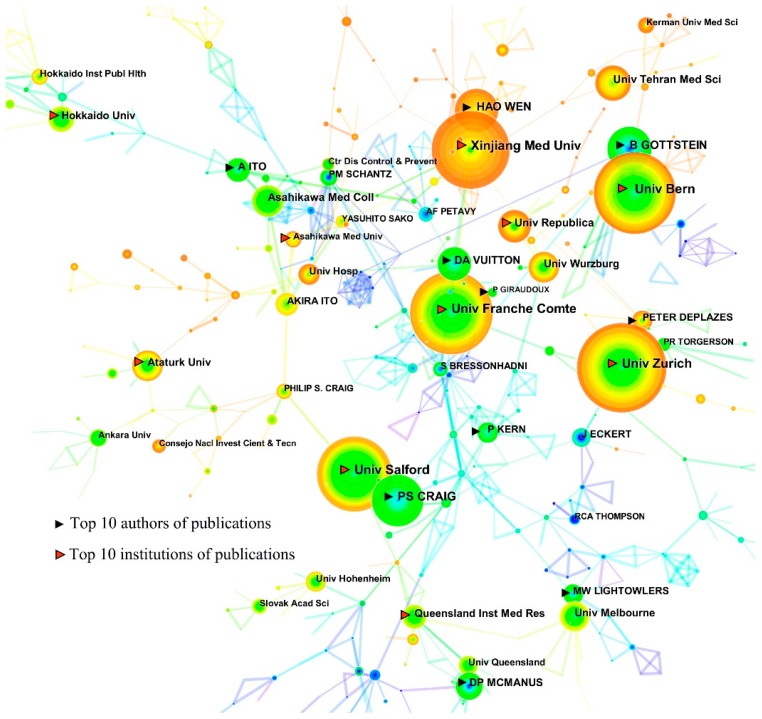
The collaboration network map of the most productive authors and institutions contributed to publications on echinococcosis research from 1980 to 2017.

The collaboration network map of the most productive authors and institutions on echinococcosis research from 1980 to 2017 is shown in Figure 4. The size of the circles represents the amount of publications and the line number between two circles suggests more collaboration between individual authors and/or institutions. Compared with institutions of the most productive authors, there was very little cooperation between the institutions (Figure 4). As can be seen from Figure 4, there are five research institutions of the most productive authors including Univ Zurich (Deplazes P), Univ Bern (Giraudoux P), Univ Salford (Craig PS), Univ Franche Comte (Vuitton DA and Giraudoux P), and Xinjiang Med Univ (Wen H). The data indicated that the major contributions on echinococcosis research were from those five institutions of the most productive authors.

### 3.4. Analysis of Co-Cited References

The analysis of references is one of the most significant indicators of bibliometrics. The co-citation map of references estimated the scientific relevance of the publications. According to co-citation counts and the centrality of references over the past 38 years, an analysis showed that such topic data are generally in the form of (1) diagnosis and treatment of echinococcosis, (2) epidemiology and clinical aspects of echinococcosis, (3) global socioeconomic impact of echinococcosis, (4) molecular phylogeny and transmission of echinococcosis, (5) prevention and surgical therapy of echinococcosis [1,33,34,35,36,37,38,39,40,41,]. 

**Figure 5:**
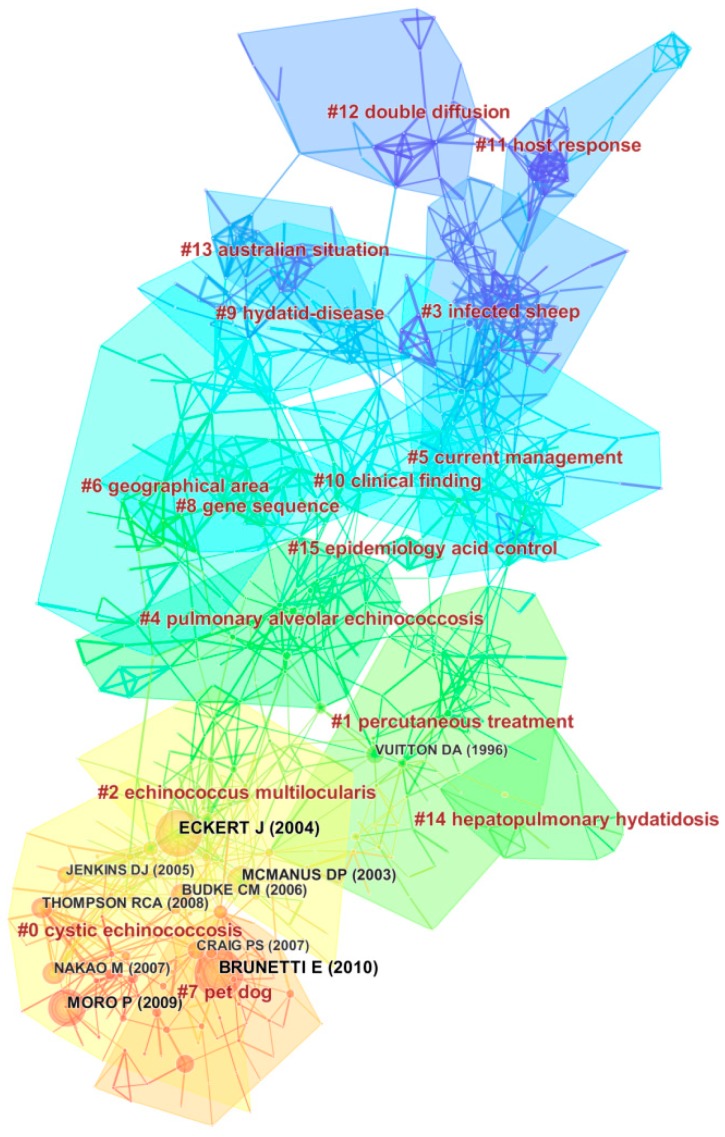
Reference co-citation map of publications on echinococcosis research from 1980 to 2017. The network is divided into 16 co-citation clusters. All clusters were labeled by appropriate index terms extracted from the references.

Generating a cited reference map resulted in 817 nodes and 1645 links with a mean Silhouette, S = 0.51 and modularity, Q = 0.81 (Figure 5). In this map, the modularity Q score was greater than 0.7, which means the network was reasonably divided into loosely coupled clusters. A cluster analysis of document co-citation was used to mine the research patterns, emerging trends and their interconnection in the echinococcosis research field. All clusters were labeled by appropriate index terms extracted from the references. To describe the nature of a cluster, noun phrases citing the cluster was extracted from the titles of publications by CiteSpace software based on three specialized log-likelihood tests (LLR), metrics-TFIDF and mutual information tests (MI) [22]. In this study, to generate high-quality clusters [22], the LLR clustering technique was used and the network was divided into 16 co-citation clusters (Figure 5). The detailed information about the top 6 clusters is summarized in a supplemental file. 

The largest cluster (#0), labeled as “cystic echinococcosis” by LLR has 83 members and a silhouette value of 0.91. The most active citer to this cluster is Omer et al. [42], “A molecular survey of cystic echinococcosis in Sudan”, which focuses on the prevalence survey of cystic echinococcosis with a PCR system in livestock in Sudan. This paper provides a foundation for future large-scale studies of the epidemiology and ecology of *E. granulosus* in developing countries, and also reflected that the researcher interests on echinococcosis were in cluster #0 in generally. The second largest cluster (#1), labeled as “percutaneous treatment” by LLR, has 78 members and a S-value of 0.90. The most active citer to this cluster is Akhan et al. [43], “percutaneous treatment of liver hydatid cysts”. This paper discussed and reviewed contraindications, indications, complications, healing criteria, method and techniques, importance and results of the percutaneous treatment of liver hydatid cysts. The third largest cluster (#2) is “*Echinococcus multilocularis*” which has 72 members and a silhouette value of 0.87. The most active citer to this cluster is Deplazes et al. [44], “veterinary aspects of alveolar echinococcosis: a zoonosis of public health significance”. They discussed the transmission and epidemiological situation in definitive hosts of wild and domestic animals. All possible comprehensive measures for preventing *E. multilocularis* infections in domestic animals and in humans should be initiated by veterinary and health authorities. The 4th largest cluster (#3) has 68 members and a silhouette value of 0.91. It is labeled as infected sheep. The most active citer to this cluster is Münst, et al. [45], “plasma-concentrations of mebendazole during treatment of echinococcosis: preliminary-results”. They found that systemic bioavailability of mebendazole is enhanced by concomitant food intake of a fatty meal for the treatment of human alveolar and cystic echinococcosis. 

There are other clusters in Figure 5. Among all clusters, cluster #7 is worth mentioning, in which the first ranked burst document was published by Brunetti et al. [41] with bursts of 108.29. The core of this article was to reach a new expert consensus for the treatment and diagnosis of human echinococcosis, which represents the emerging trends and active fields. The consensus of experts under the aegis of the WHO-IWGE would help promote echinococcosis studies of the missing evidence field. The second ranked burst document was published by Eckert et al. with bursts of 108.40 in cluster #2 [36]. This article focused on biological, epidemiological and clinical aspects, including the emergence or re-emergence of infections in regions where they were found at lower levels or were previously absent. The third ranked burst document was published by Moro et al. with bursts of 73.68 in cluster #0 [46]. This work discussed the pathogen, distribution, and transmission of the *E.* organisms, and epidemiology, clinical features, laboratory findings and diagnosis, treatment including monitoring results of treatment of the diseases. New specific and sensitive diagnostic methods and effective therapeutic measures against echinococcosis have been developed from 1998 to 2008. 

### 3.5. Co-Occurring Keywords Analysis

The knowledge map of keyword co-occurrence can suggest hot topics and burst keywords can reflect frontier topics [17,25]. In the current study, keywords that occurred in the 7688 publications were extracted and analyzed with CiteSpace. Generating a keyword co-occurrence map using CiteSpace resulted in 109 nodes and 503 links with a mean Silhouette, S = 0.80 and modularity, Q = 0.79. Since the Q-value of the keywords network is under the average of 0.70, the nodes within the keywords network map are densely packed (data not present). The keywords with over 400 usage count are identified in a supplemental file and the top five keywords were as follows: *Echinococcosis granulosus* (1120 counts), Hydatid cyst (991 counts), Liver (761 counts), Diagnosis (695 counts) and Cystic echinococcosis (660 counts). The analytical data in terms of co-occurrence frequency and centrality displayed that the hot keywords were *Echinococcus granulosus* (cystic echinococcosis), diagnosis, epidemiology (prevalence), *Echinococcus multiloculari* (alveolar echinococcosis), and treatment (albendazole etc.).

**Figure 6:**
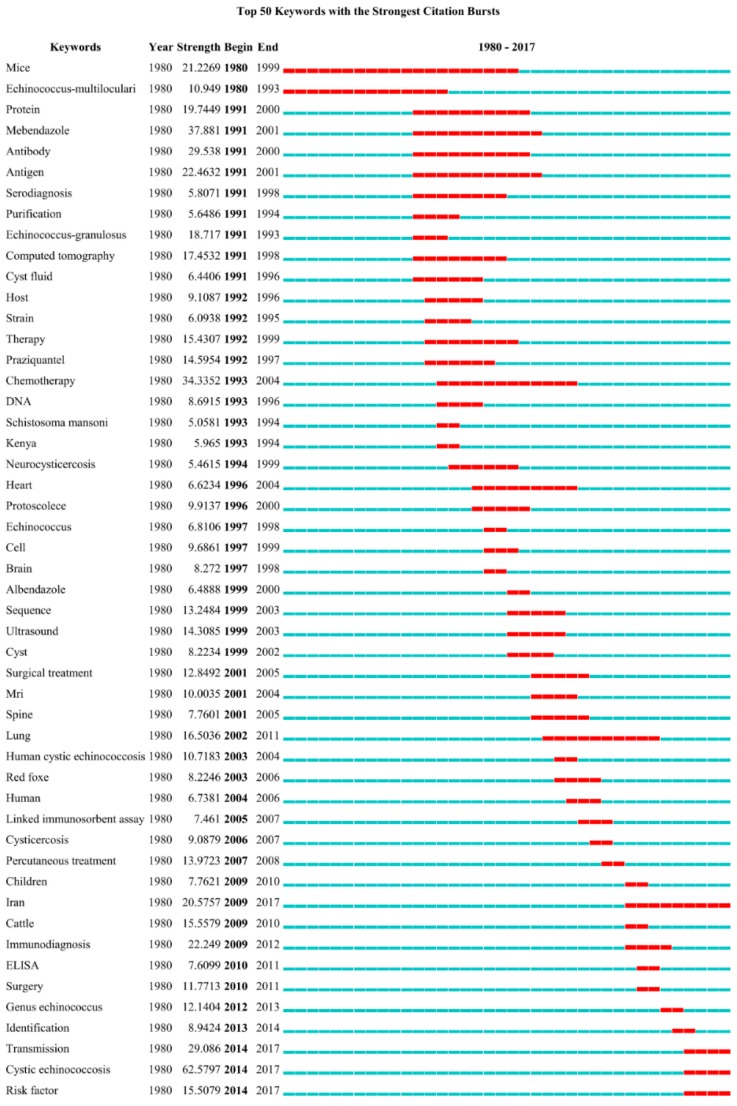
Keywords with the strongest citation bursts of publications on echinococcosis research from 1980 to 2017.

The keywords with the strongest citation bursts were also detected and analyzed with CiteSpace (Figure 6). The keyword of cystic echinococcosis (62.6) was the first-class strongest burst keyword, which was during the period between 2014 and 2017. The keyword of mebendazole (37.9) was the second-class strongest burst keyword during the period between 1992 and 2006. The keyword of antibody (29.5) was the third-class strongest burst keyword during the period between 1992 and 2006. The keyword of transmission during the period between 2014 and 2017 was the fourth-class strongest burst keyword. According to the top 50 strongest burst keywords and cluster analysis of document co-citation of echinococcosis research, we inferred the top four research hot topics and emerging trends which are listed as follows.

#### 3.5.1. Cystic Echinococcosis

CE is the most common performance and caused by *E. granulosus* worldwide [47]. The most interesting research is that scientists discovered *E. granulosus* exhibits considerable variation in terms of host range, morphology, infectivity to humans and pathogenicity [48]. According to DNA sequences of all protein-coding genes from mitochondrial genomes, at least eight distinct genotypes (G1, G3, G4-G8; and G10) or strains have been characterized and classified which included *E. granulosus sensu stricto* (s.s.) (genotype G1, G3), *Echinococcus equinus* (genotype G4), *Echinococcus ortleppi* (genotype G5), *Echinococcus canadensis* (genotypes G6-G8, G10), and the lion strain *Echinococcus felidis* [48,49,50,51,]. Genotypes G9 and G2 are currently treated as invalid: G9 is a variant of G7 and G2 belongs to the genotype G3 cluster [49]. Genotype G1 is sheep strains, while genotype G3 and G5 are buffalo and cattle strains, respectively. Genotype G4 is found in horses, genotype G6 in camels, genotype G7 in pigs and genotype G8/G10 in cervids [52,53]. These genotypes such as G1, G3, G4, G5 are now regarded as distinct species, the status of genotypes G6–G9 and G10 is still under dispute [48,53], and some of that remains unknown [50]. The accurate genotype has an important epidemiological implication in endemic regions and clues to the zoonotic potential of particular genotypes [48]. Studying these genetic variations and polymorphisms in *E. granulosus* populations is meaningful for better understanding of the various life cycles of CE and shedding light on more efficient control and prevention strategies.

#### 3.5.2. Diagnosis

Diagnosis of echinococcosis is mainly confirmed through a combination of relevant history and serological testing, along with imaging approaches of ultrasonography, computer tomography (CT) or/and magnetic resonance imaging (MRI) [54]. The differential diagnosis (liver tumors, abscess etc.) of echinococcosis at the early stage was difficult in many cases [50]. Over recent years, attempts were made to introduce and apply improved diagnostic methods. A variety of serum immunological examinations such as the latex agglutination test, indirect fluorescent-antibody (IFA), enzyme-linked immunosorbent assays (ELISA), immunochromatography test and immunoblotting were developed and used to detect specific antibodies for supporting the clinical diagnosis or epidemiological studies on echinococcosis in recent years [55]. However, these immunological methods have been challenging due to cross-reactivity problems with other parasitic antigens or with non-parasitic diseases [56,57]. The newer diagnostic methods on serological, molecular, and proteomic approaches must be developed in the future.

#### 3.5.3. Chemotherapy

Four treatment options for CE have been recommended based on the WHO-IWGE classification of cyst stages as seen in ultrasonography-based imaging findings [58]: observation (watch-and-wait approach) for inactive, clinically silent cysts, chemotherapy with benzimidazoles, percutaneous sterilization and surgery [59]. There are three relevant groups of cysts: active (CE1 and 2), transitional (CE3a and 3b) and inactive (CE4 and 5). CE1 and CE3a are early stages and CE4 and CE5 late stages [41]. The classification of cysts is used for staging of the treatment selection [41]. Surgery, chemotherapy and percutaneous treatments are usually not indicated in uncomplicated inactive cysts, but rather demand for the active (CE1 and 2) and transitional (CE3a and 3b) cysts [41]. Each of these treatment options has limitations depending on the individual case. Moreover, the clinical study evidence of these therapeutic tools is insufficient and the choice of therapeutic options remains controversial [60]. Surgical operation removal is not applicable for cases with multiple cysts in two or more organs, in patients for pre-surgical treatment and for prevention of secondary echinococcosis after surgery [61]. Chemotherapy of echinococcosis with mebendazole or albendazole is often only partially effective, and rarely curative with complete regression of the cysts [41]. Therefore, it is necessary to develop novel compounds and/or more efficient chemotherapy treatment options.

#### 3.5.4. Transmission of Animals

Regional differences in haplotype diversity led to a hypothesis on the origin of *E. granulosus* in a wildlife cycle in those regions. While definitive hosts are most commonly dogs and wild carnivores, a wide range of domestic and wild mammals, but also humans, act as intermediate or accidental hosts [48,62]. AE is usually maintained by the sylvatic cycle (fox/rodents), which can be linked with domestic cats and dogs [48,62]. CE is mainly supported by a domestic cycle (dog/domestic ungulate), which can persist in rural livestock-raising areas where humans cohabit with dogs fed on raw livestock offal [2]. Controlling the infection of parasites in animals is crucial to limit the transmission and reduce the incidence of human disease. In order to design the most effective control programs for reducing transmission to humans, the study of echinococcosis epidemiology on animal hosts has positive significance.

## 4. Conclusions

In conclusion, visualized network and co-citation analysis of the reference on the echinococcosis research field were firstly calculated with software of CiteSpace. The top three productive countries were Turkey (1133 articles), France (582 articles), China (574 articles), and France, China, USA, England, Australia and Switzerland were the major research powers in the echinococcosis field. The top three productive institutions were the Univ Zurich (German, 229 articles), Univ Bern (Switzerland, 203 articles) and Univ Salford (England, 173 articles). The top three productive scientists were Craig PS (UK, 171 articles), Gottstein B (Switzerland, 167 articles) and Wen H (China, 126 articles) on echinococcosis research. The top three productive journals were respectively the Veterinary Parasitology, Parasitology Research, and Parasitology. CE ranked the first in research hotspots, chemotherapy and immunological diagnosis of CE, management of definitive and intermediate host listed as the first in research frontiers. 

Though many intervention programmes were adopted and the transmission of *E. granulosus* and *E. multilocularis* can be controlled effectively in both island and continental settings in the early part of the 21st century [63], prevention and control of global echinococcosis is still challenged especially when treatment of humans has no ability to interrupt transmission. Since humans cannot transmit CE (or AE), human treatment does not play a crucial role in control programs for these two zoonoses. In addition to the effects of echinococcosis on livestock, health issues are chronic, and livestock, fox and dog hosts are generally asymptomatic. Furthermore, multifaceted wildlife-human interactions may affect population dynamics of final and intermediate host communities. A number of intervention approaches of echinococcosis remains to be undertaken. 

However, in this study, it is extremely difficult to gain an entire picture of the echinococcosis research field due to the complicacy of echinococcosis. Compared with the literature discussion from domain experts, the analysis with CiteSpace software in the current study could be shallow and controversial to some extent. Regardless, we have utilized a quantitative statistical analysis tool to investigate firstly the knowledge progress of the echinococcosis domain by literature mining strategies, which can help us understand the patterns and trends in the echinococcosis field visually.

## Supplementary Materials

The following are available online at https://www.mdpi.com/1660-4601/16/5/842/s1, Table S1: Top 10 countries contributed to publications on echinococcosis research from 1980 to 2017, Table S2: Top 10 institutions contributed to publications on echinococcosis research from 1980 to 2017, Table S3: Top 10 authors of publications and cited authors on echinococcosis research in terms of co-citation counts and centrality from 1980 to 2017, Table S4: Top 10 journals of publications and cited journals on echinococcosis research in terms of co-citations counts and centrality from 1980 to 2017, Table S5: Top 10 co-cited references related to echinococcosis research from 1980 to 2017, Table S6: The 6 top-ranked clusters in the echinococcosis field from 1980 to 2017, Table S7: Top 10 keywords of publications on echinococcosis research in terms of citation counts and centrality.

## References

[1] Craig P.S., McManus D.P., Lightowlers M.W., Chabalgoity J.A., Garcia H.H., Gavidia C.M., Gilman R.H., Gonzalez A.E., Lorca M., Naquira C. (2007). Prevention and control of cystic echinococcosis. Lancet Infect. Dis..

[2] Otero-Abad B., Torgerson P.R. (2013). A systematic review of the epidemiology of echinococcosis in domestic and wild animals. PLoS Negl. Trop. Dis..

[3] Khademvatan S., Yousefi E., Rafiei A., Rahdar M., Saki J. (2013). Molecular characterization of livestock and human isolates of *Echinococcus granulosus* from south-west Iran. J. Helminthol..

[4] Cucher M.A., Macchiaroli N., Baldi G., Camicia F., Prada L., Maldonado L., Avila H.G., Fox A., Gutiérrez A., Negro P. (2016). Cystic echinococcosis in South America: Systematic review of species and genotypes of *Echinococcus granulosus sensu lato* in humans and natural domestic hosts. Trop. Med. Int. Health.

[5] Bartsch S.M., Hotez P.J., Asti L., Zapf K.M., Bottazzi M.E., Diemert D.J., Lee B.Y. (2016). The global economic and health burden of human hookworm infection. PLoS Negl. Trop. Dis..

[6] Lahmar S., Chéhida F.B., Pétavy A.F., Hammou A., Lahmar J., Ghannay A., Gharbi H.A., Sarciron M.E. (2007). Ultra-sonographic screening for cystic echinococcosis in sheep in Tunisia. Vet. Parasitol..

[7] Torgerson P.R., Keller K., Magnotta M., Ragland N. (2010). The global burden of alveolar echinococcosis. PLoS Negl. Trop. Dis..

[8] Rahimi-Esboei B., Ebrahimzadeh M.A., Fathi H., Rezaei Anzahaei F. (2016). Scolicidal effect of allium sativum flowers on hydatid cyst protoscolices. Eur. Rev. Med. Pharmacol. Sci..

[9] Torgerson P.R., Oguljahan B., Muminov A.E., Karaeva R.R., Kuttubaev O.T., Aminjanov M., Shaikenov B. (2006). Present situation of cystic echinococcosis in Central Asia. Parasitol. Int..

[10] Grosso G., Gruttadauria S., Biondi A., Marventano S., Mistretta A. (2012). Worldwide epidemiology of liver hydatidosis including the Mediterranean area. World J. Gastroenterol..

[11] Mehta P., Prakash M., Khandelwal N. (2016). Radiological manifestations of hydatid disease and its complications. Trop. Parasitol..

[12] Torgerson P.R. (2013). The emergence of echinococcosis in central Asia. Parasitology.

[13] Zhang W., Zhang Z., Wu W., Shi B., Li J., Zhou X., Wen H., McManus D.P. (2015). Epidemiology and control of echinococcosis in central Asia; with particular reference to the People’s Republic of China. Acta Trop..

[14] Royle P., Kandala N.B., Barnard K., Waugh N. (2013). Bibliometrics of systematic reviews: Analysis of citation rates and journal impact factors. Syst. Rev..

[15] Leefmann J., Levallois C., Hildt E. (2016). Neuroethics 1995–2012. A bibliometric analysis of the guiding themes of an emerging research field. Front. Hum. Neurosci..

[16] Ma R., Ho Y.S. (2016). Comparison of environmental laws publications in Science Citation Index Expanded and Social Science Index: A bibliometric analysis. Scientometrics.

[17] Chen C. (2006). CiteSpace II: Detecting and visualizing emerging trends and transient patterns in scientifc literature. J. Am. Soc. Inf. Sci. Technol..

[18] Liu Z., Yin Y., Liu W., Dunford M. (2015). Visualizing the intellectual structure and evolution of innovation systems research: A bibliometric analysis. Scientometrics.

[19] Lee Y.C., Chen C.M., Tsai X.T. (2016). Visualizing the knowledge domain of nanoparticle drug delivery technologies: A scientometric review. Appl. Sci..

[20] Liang Y.D., Li Y., Zhao J., Wang X.Y., Zhu H.Z., Chen X.H. (2017). Study of acupuncture for low back pain in recent 20 years: A bibliometric analysis via CiteSpace. J. Pain Res..

[21] Zongyi Y., Dongying C., Baifeng L. (2017). Correction: Global regulatory T-cell research from 2000 to 2015: A bibliometric analysis. PLoS ONE.

[22] Xiao F., Li C., Sun J., Zhang L. (2017). Knowledge domain and emerging trends in organic photovoltaic technology: A scientometric review based on citespace analysis. Front. Chem..

[23] Miao Y., Liu R., Pu Y., Yin L. (2017). Trends in esophageal and esophagogastric junction cancer research from 2007 to 2016 a bibliometric analysis. Medicine.

[24] Chen C. (2004). Searching for intellectual turning points: Progressive knowledge domain visualization. Proc. Natl. Acad. Sci. USA.

[25] Kleinberg J. (2002). Bursty and hierarchical structure in streams. Data Min. Knowl. Dis..

[26] Li M.N., Porter A.L., Wang Z.L. (2017). Evolutionary trend analysis of nanogenerator research based on a novel perspective of phased bibliographic coupling. Nano Energy.

[27] Giraudoux P., Raoul F., Pleydell D., Craig P.S. (2008). Multidisciplinary studies.; systems approaches and parasite eco-epidemiology: Something old.; something new. Parasite.

[28] Casulli A., Manfredi M.T., La Rosa G., Cerbo A.R., Genchi C., Pozio E. (2008). *Echinococcus* ortleppi and *E. granulosus* G1, G2 and G3 genotypes in Italian bovines. Vet. Parasitol..

[29] Li T., Ito A., Nakaya K., Qiu J., Nakao M., Zhen R., Xiao N., Chen X., Giraudoux P., Craig P.S. (2008). Species identification of human echinococcosis using histopathology and genotyping in northwestern China. Trans. R. Soc. Trop. Med. Hyg..

[30] Eckert J., Thompson R.C. (2017). Historical aspects of echinococcosis. Adv. Parasitol..

[31] Thompson R.C.A., Ash A. (2016). Molecular epidemiology of Giardia and Cryptosporidium infections. Infect. Genet. Evol..

[32] McManus D.P., Smyth J.D. (1982). Intermediary carbohydrate metabolism in protoscoleces of *Echinococcus granulosus* (horse and sheep strains) and *E. multilocularis*. Parasitology.

[33] Vuitton D.A., Meslin F.X., Eckert J., Writing Panel for the WHO-IWGE (1996). Guidelines for treatment of cystic and alveolar echinococcosis in humans. Bull. World Health Organ..

[34] Khuroo M.S., Wani N.A., Javid G., Khan B.A., Yattoo G.N., Shah A.H., Jeelani S.G. (1997). Percutaneous drainage compared with surgery for hepatic hydatid cysts. N. Engl. J. Med..

[35] Kern P., Bardonnet K., Renner E., Auer H., Pawlowski Z., Ammann R.W., Vuitton D.A., Kern P., European Echinococcosis Registry (2003). European echinococcosis registry: Human alveolar echinococcosis, Europe, 1982–2000. Emerg. Infect. Dis..

[36] Eckert J., Deplazes P. (2004). Biological, epidemiological and clinical aspects of echinococcosis, a zoonosis of increasing concern. Clin. Microbiol. Rev..

[37] Budke C.M., Deplazes P., Torgerson P.R. (2006). Global socioeconomic impact of cystic echinococcosis. Emerg. Infect. Dis..

[38] Nakao M., McManus D.P., Schantz P.M., Craig P.S., Ito A. (2007). A molecular phylogeny of the genus *Echinococcus* inferred from complete mitochondrial genomes. Parasitology.

[39] Schweiger A., Ammann R.W., Candinas D., Clavien P.A., Eckert J., Gottstein B., Halkic N., Muellhaupt B., Prinz B.M., Reichen J. (2007). Human alveolar echinococcosis after fox population increase, Switzerland. Emerg. Infect. Dis..

[40] Thompson R.C. (2008). The taxonomy, phylogeny and transmission of *Echinococcus*. Exp. Parasitol..

[41] Brunetti E., Kern P., Vuitton D.A., Writing Panel for the WHO-IWGE (2010). Expert consensus for the diagnosis and treatment of cystic and alveolar echinococcosis in humans. Acta Trop..

[42] Omer R.A., Dinkel A., Romig T., Mackenstedt U., Elnahas A.A., Aradaib I.E., Ahmed M.E., Elmalik K.H., Adam A. (2010). A molecular survey of cystic echinococcosis in Sudan. Vet. Parasitol..

[43] Akhan O., Ozmen M.N. (1999). Percutaneous treatment of liver hydatid cysts. Eur. J. Radiol..

[44] Deplazes P., Eckert J. (2001). Veterinary aspects of alveolar echinococcosis—A zoonosis of public health significance. Vet. Parasitol..

[45] Münst G.J., Karlaganis G., Bircher J. (1980). Plasma-concentrations of mebendazole during treatment of echinococcosis—Preliminary-results. Eur. J. Clin. Pharmacol..

[46] Moro P., Schantz P.M. (2009). Echinococcosis: A review. Int. J. Infect. Dis..

[47] Wu C., Zhang W., Ran B., Fan H., Wang H., Guo B., Zhou C., Shao Y., Zhang W., Giraudoux P. (2017). Genetic variation of mitochondrial genes among *Echinococcus multilocularis* isolates collected in western China. Parasite Vectors.

[48] Kinkar L., Laurimäe T., Balkaya I., Casulli A., Zait H., Irshadullah M., Sharbatkhori M., Mirhendi H., Rostami-Nejad M., Ponce-Gordo F. (2018). Genetic diversity and phylogeography of the elusive.; but epidemiologically important *Echinococcus granulosus sensu stricto* genotype G3. Parasitology.

[49] Laurimäe T., Kinkar L., Romig T., Omer R.A., Casulli A., Umhang G., Gasser R.B., Jabbar A., Sharbatkhori M., Mirhendi H. (2018). The benefits of analysing complete mitochondrial genomes: Deep insights into the phylogeny and population structure of *Echinococcus granulosus sensu lato* genotypes G6 and G7. Infect. Genet. Evol..

[50] Deplazes P., Rinaldi L., Alvarez Rojas C.A., Torgerson P.R., Harandi M.F., Romig T., Antolova D., Schurer J.M., Lahmar S., Cringoli G. (2017). Global distribution of alveolar and cystic echinococcosis. Adv. Parasit..

[51] Ito A., Budke C.M. (2017). The echinococcoses in Asia: The present situation. Acta Trop..

[52] Alvarez Rojas C.A., Romig T., Lightowlers M.W. (2014). *Echinococcus granulosus sensu lato* genotypes infecting humans—Review of current knowledge. Int. J. Parasitol..

[53] Khademvatan S., Majidiani H., Foroutan M., Hazrati Tappeh K., Aryamand S., Khalkhali H.R. (2018). *Echinococcus granulosus* genotypes in Iran: A systematic review. J. Helminthol..

[54] Keong B., Wilkie B., Sutherland T., Fox A. (2018). Hepatic cystic echinococcosis in Australia: An update on diagnosis and management. A. N. Z. J. Surg..

[55] Maurelli M.P., Bosco A., Pepe P., Ianniello D., Amadesi A., Cringoli G., Rinaldi L. (2018). Innovative tools for the diagnosis of *Echinococcus granulosus* in definitive hosts. Parasitol. Res..

[56] Carmena D., Benito A., Eraso E. (2006). Antigens for the immunodiagnosis of *Echinococcus granulosus* infection: An update. Acta Trop..

[57] Mihmanli M., Idiz U.O., Kaya C., Demir U., Bostanci O., Omeroglu S., Bozkurt E. (2016). Current status of diagnosis and treatment of hepatic echinococcosis. World J. Hepatol..

[58] Siles-Lucas M., Casulli A., Cirilli R., Carmena D. (2018). Progress in the pharmacological treatment of human cystic and alveolar echinococcosis: Compounds and therapeutic targets. PLoS Negl. Trop. Dis..

[59] Brunetti E., Garcia H.H., Junghanss T., International C.E. (2011). Workshop in Lima, Peru, 2009. Cystic chinococcosis: Chronic, complex, and still neglected. PLoS Negl. Trop. Dis..

[60] Stojkovic M., Zwahlen M., Teggi A., Vutova K., Cretu C.M., Virdone R., Nicolaidou P., Cobanoglu N., Junghanss T. (2009). Treatment response of cystic echinococcosis to benzimidazoles: A systematic review. PLoS Negl. Trop. Dis..

[61] Nunnari G., Pinzone M.R., Gruttadauria S., Celesia B.M., Madeddu G., Malaguarnera G., Pavone P., Cappellani A., Cacopardo B. (2012). Hepatic echinococcosis: Clinical and therapeutic aspects. World J. Gastroenterol..

[62] Romig T., Deplazes P., Jenkins D., Giraudoux P., Massolo A., Craig P.S., Wassermann M., Takahashi K., de la Rue M. (2017). Ecology and Life Cycle Patterns of *Echinococcus* Species. Adv. Parasitol..

[63] Craig P.S., Hegglin D., Lightowlers M.W., Torgerson P.R., Wang Q. (2017). Echinococcosis: Control and prevention. Adv. Parasitol..

