# Supplementary material for: Knowledge Domain and Emerging Trends on Echinococcosis Research: A Scientometric Analysis
Source: Int J Environ Res Public Health. 2019 Mar 8;16(5):842. doi: 10.3390/ijerph16050842 (PMC6427242; doi:10.3390/ijerph16050842)
Supplement: Supplementary file 1 [file ijerph-16-00842-s001.pdf]

International Journal of
Environmental Research and Public Health

MDPI

Communication

Knowledge Domain and Emerging Trends on Echinococcosis Research: A Scientometric Analysis

Supplemental data

Table S1. Top 10 countries contributed to publications on echinococcosis research from 1980 to 2017.

Rank	Country	Count	Centrality	Percent (%)
1	TURKEY	1,133	0.00	14.74%
2	FRANCE	582	0.30	7.57%
3	PEOPLES R CHINA	574	0.56	7.47%
4	USA	521	0.56	6.78%
5	ENGLAND	485	0.44	6.31%
6	GERMANY	482	0.14	6.27%
7	SWITZERLAND	480	0.64	6.24%
8	INDIA	376	0.00	4.89%
9	AUSTRALIA	356	0.34	4.63%
10	SPAIN	355	0.00	4.62%

International Journal of
Environmental Research and Public Health

MDPI

Table S2. Top 10 institutions contributed to publications on echinococcosis research from 1980 to 2017.

Rank	Institution	Count	Centrality	Percent (%)
1	Univ Zurich (German)	229	0.14	2.98%
2	Univ Bern (Switzerland)	203	0.14	2.64%
3	Univ Salford (England)	173	0.23	2.25%
4	Univ Franche Comte (France)	166	0.22	2.16%
5	Xinjiang Med Univ (China)	150	0.04	1.95%
6	Univ Republica (Spanish)	116	0.03	1.51%
7	Ataturk Univ (Turkish)	102	0.10	1.33%
8	Hokkaido Univ (Japan)	96	0.04	1.25%
9	Queensland Inst Med Res (Australia)	79	0.03	1.03%
10	Asahikawa Med Coll (Japan)	78	0.03	1.02%

Table S3. Top 10 authors of publications and cited authors on echinococcosis research in terms of co-citation counts and centrality from 1980 to 2017.

Rank	Publications			Co-citations		Centrality	
	Author/ Country	Count	Percent (%)	Citation counts	Cited author	Centrality	Cited author
1	Craig PS/ UK	171	2.22%	1508	Eckert J, 1980	0.54	Beard TC, 1980
2	Gottstein B/ Switzerland	167	2.17%	907	Thompson RCA, 1982	0.54	Nelson GS, 1981
3	Wen H/ China	126	1.64%	787	Craig PS, 1984	0.49	Eckert J, 1980
4	Vuitton DA/ France	123	1.60%	783	Mcmanus DP, 1982	0.39	Schwabe CW, 1982
5	Ito A/ Japan	120	1.56%	751	Gottstein B, 1984	0.36	Kern P, 1980
6	Deplazes P/ Switzerland	113	1.47%	661	Schantz PM, 1980	0.36	Purriel P, 1983
7	Mcmanus DP/ Australia	109	1.42%	587	Vuitton DA, 1988	0.33	Mcmanus DP, 1982
8	Kern P/ Germany	94	1.22%	486	Deplazes P, 1994	0.31	Gemmell MA, 1980
9	Lightowlers MW/ Australia	79	1.03%	462	Torgerson PR, 2003	0.30	Verster AJM, 1982
10	Giraudoux P/ France	77	1.00%	421	Rausch RL, 1980	0.29	Farag H, 1980

International Journal of
Environmental Research and Public Health

MDPI

Table S4. Top 10 journals of publications and cited journals on echinococcosis research in terms of co-citations counts and centrality from 1980 to 2017.

Rank	Publications			Co-citations		Centrality	
	Journals	Count	Percent (%)	Citation counts	Cited journal	Centrality	Cited journal
1	Vet Parasitol	260	3.38%	2245	Parasitology	0.78	Nature
2	Parasitol Res	250	3.25%	2202	Int J Parasitol	0.71	Clin Exp Immunol
3	Parasitology	180	2.34%	2201	Am J Trop Med Hyg	0.71	Immunology
4	Acta Trop	161	2.09%	1846	Acta Trop	0.71	Tropenmed Parasitol
5	Am J Trop Med Hyg	147	1.91%	1593	Parasitol Res	0.69	J Exp Med
6	Int J Parasitol	145	1.89%	1561	Vet Parasitol	0.67	Acta Trop
7	J Helminthol	125	1.63%	1415	T Roy Soc Trop Med H	0.66	Int J Parasitol
8	Ann Trop Med Parasit	124	1.61%	1409	Ann Trop Med Parasit	0.65	Brit Med J
9	Parasitol Int	105	1.37%	1257	J Parasitol	0.65	Acta Gastro-Ent Belg
10	Parasite Immunol	104	1.35%	2245	Mol Biochem Parasit	0.64	Med J Australia

International Journal of
Environmental Research and Public Health

MDPI

Table S5. Top 10 co-cited references related to echinococcosis research from 1980 to 2017.

Rank	Co-citation counts	Centrality	Cited reference	Author/publication year/ journal
1	264	0.02	Expert consensus for the diagnosis and treatment of cystic and alveolar echinococcosis in humans	Brunetti E, 2010, Acta Trop (Brunetti et al. 2010)
2	255	0.03	Biological, epidemiological, and clinical aspects of echinococcosis, a zoonosis of increasing concern	Eckert J, 2004, Clin Microbiol Rev (Eckert et al. 2004)
3	130	0.02	A molecular phylogeny of the genus Echinococcus inferred from complete mitochondrial genomes	Nakao M, 2007, Parasitology (Nakao et al. 2007)
4	128	0.05	The taxonomy, phylogeny and transmission of Echinococcus	Thompson RCA, 2008, Exp Parasitol (Thompson 2008)
5	121	0.05	Global socioeconomic impact of cystic echinococcosis	Budke CM, 2006, Emerg Infect Dis (Budke et al. 2006)
6	105	0.01	Guidelines for treatment of cystic and alveolar echinococcosis in humans	Vuitton DA, 1996, B World Health Organ (Vuitton et al. 1996)
7	105	0.12	Prevention and control of cystic echinococcosis	Craig PS, 2007, Lancet Infect Dis (Craig et al. 2007)
8	88	0.06	Human alveolar echinococcosis after fox population increase	Schweiger A, 2007, Emerg Infect Dis (Schweiger et al. 2007)
9	77	0.04	European echinococcosis registry: human alveolar echinococcosis, Europe, 1982-2000	Kern P, 2003 , Emerg Infect Dis (Kern et al. 2003)
10	72	0.17	Percutaneous drainage compared with surgery for hepatic hydatid cysts	Khuroo MS, 1997, New Engl J Med (Khuroo et al. 1997)

Table S6. The 6 top-ranked clusters in the echinococcosis field from 1980 to 2017.

Cluster ID	Size	Silhouette	Mean (cite year)	Label (TFIDF)	Label (LLR)	Label (MI)
#0	83	0.909	2008	echinococcus granulosus	cystic echinococcosis	tomographic diagnosis
#1	78	0.897	1995	liver	percutaneous treatment	comparative proteome profiling
#2	72	0.872	2001	echinococcus multilocularis	echinococcus multilocularis	hydatid fluid
#3	68	0.909	1978	mebendazole	infected sheep	Algerian patient
#4	58	0.793	1993	alveolar echinococcosis	pulmonary alveolar echinococcosis	major cyst fluid antigen
#5	54	0.857	1984	hydatid-disease	current management	liver echinococcosis

International Journal of
Environmental Research and Public Health

MDPI

Table S7. Top 10 keywords of publications on echinococcosis research in terms of citation counts and centrality.

Rank	Citation counts		Centrality	
	Counts	Keyword	Centrality	Keyword
1	1120	Echinococcus granulosus	0.30	Echinococcus granulosus
2	991	Hydatid cyst	0.14	Alveolar echinococcosis
3	761	Liver	0.14	Epidemiology
4	695	Diagnosis	0.11	Albendazole
5	660	Cystic echinococcosis	0.1	Prevalence
6	619	Echinococcus multiloculari	0.1	Diagnosis
7	574	Cyst	0.1	Albendazole
8	550	Alveolar echinococcosis	0.09	Echinococcus multiloculari
9	510	Albendazole	0.08	Cystic echinococcosis
10	400	Prevalence	0.08	Liver

© 2019 by the authors. Submitted for possible open access publication under the terms and conditions of the Creative Commons Attribution (CC BY) license (http://creativecommons.org/licenses/by/4.0/).
